# Direct visualization of current-induced spin accumulation in topological insulators

**DOI:** 10.1038/s41467-018-04939-6

**Published:** 2018-06-27

**Authors:** Yang Liu, Jean Besbas, Yi Wang, Pan He, Mengji Chen, Dapeng Zhu, Yang Wu, Jong Min Lee, Lan Wang, Jisoo Moon, Nikesh Koirala, Seongshik Oh, Hyunsoo Yang

**Affiliations:** 1 Department of Electrical and Computer Engineering, National University of Singapore, Singapore, 117576 Singapore; 2 RMIT University, School of Science, Department of Physics, Melbourne, VIC, 3001 Australia; 3 Department of Physics and Astronomy, Rutgers, The State University of New Jersey, Piscataway, NJ 08854 USA

## Abstract

Charge-to-spin conversion in various materials is the key for the fundamental understanding of spin-orbitronics and efficient magnetization manipulation. Here we report the direct spatial imaging of current-induced spin accumulation at the channel edges of Bi_2_Se_3_ and BiSbTeSe_2_ topological insulators as well as Pt by a scanning photovoltage microscope at room temperature. The spin polarization is along the out-of-plane direction with opposite signs for the two channel edges. The accumulated spin direction reverses sign upon changing the current direction and the detected spin signal shows a linear dependence on the magnitude of currents, indicating that our observed phenomena are current-induced effects. The spin Hall angle of Bi_2_Se_3_, BiSbTeSe_2_, and Pt is determined to be 0.0085, 0.0616, and 0.0085, respectively. Our results open up the possibility of optically detecting the current-induced spin accumulations, and thus point towards a better understanding of the interaction between spins and circularly polarized light.

## Abstract (web summary)

Charge-to-spin conversion is a key theme of spintronics and its realization in topological materials is highly desired. Here, Liu et al. report spatial imaging of current-induced spin accumulation at the edges of Bi_2_Se_3_ and BiSbTeSe_2_ topological insulators.

## Introduction

Charge-to-spin conversion has been considered as one of the core research fields in spintronics^1–5^. Over the past decade, current-induced spin accumulation due to the spin Hall effect (SHE) in semiconducting systems has been extensively investigated by means of magneto-optical Kerr effects (MOKE)^6–8^, circularly polarized electroluminescence^9,10^, and two-color optical coherence techniques^11^. In metallic systems, electrical detection of current-induced spin accumulation has been studied by employing spin valve^12–14^, spin pumping^15,16^, and spin torque ferromagnetic resonance^17–19^. While the experimental situation in heavy metals for a direct optical detection of SHE is less clear with two groups suggesting a successful measurement of the SHE in Pt and W using MOKE^20,21^ and others concluding that MOKE is not suitable in measuring SHE in metallic systems^22,23^.

The three-dimensional topological insulator (TI) is a new phase of the quantum state of materials that possess spin-momentum locked surface states and insulating bulk states (BS)^24–29^. So far, the surface state-related phenomena of TIs responding to light have been extensively explored, including the chemical potential drop-assisted photovoltage generation^30–32^, circularly polarized light-induced helicity-dependent current^33^, and the circular photogalvanic effect in TIs^34^. However, the interaction between the current-induced spin accumulation with circularly polarized light is less studied especially involving the direct visualization of current-induced spin accumulation in TIs.

The spin-to-charge interconversion in TIs has been extensively studied based on ferromagnet (FM)/TI bilayer structures where the FM layer can be used as either a spin injector or detector^35–40^. In such FM/TI bilayer systems, the measured spin Hall angle could involve not only intrinsic TI contributions but also the contribution from the FM/TI interface^41–43^, which can largely modulate the effective spin Hall angle in TIs. Thus it is of great importance to quantitatively characterize the spin-to-charge conversion of TIs without involving a FM layer.

In this work, we use the scanning photovoltage microscope to image current-induced spin accumulations near channel edges in Bi_2_Se_3_ and BiSbTeSe_2_ TIs at room temperature. We further image the current-induced spin accumulation in a Pt heavy metal. Our work opens up a possibility of optically detecting the accumulated spins in various metallic and semiconducting materials, and helps to extract spin-related parameters such as the spin Hall angle and spin lifetime, propelling a better understanding of the interactions between spins and light in various materials systems.

## Results

### Principle of helicity-dependent photovoltages

**Fig. 1:**
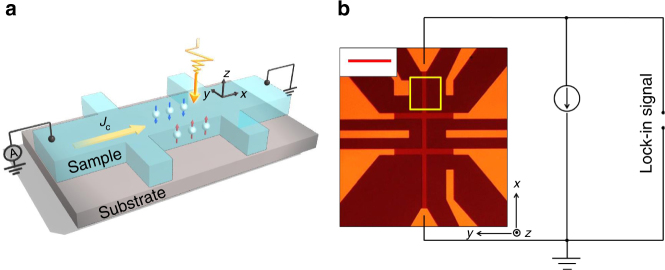
Schematics of spin-dependent photovoltage generation and experimental setup. **a** Schematic of the device structure with bias current *J*_c_ applied along the *x* direction. The laser is focused on the device with normal incidence. **b** Optical image of the device with a representation of the electrical connections. Scale bar is 50 µm. The scanning area is indicated by a yellow box. The induced photovoltage is acquired by a lock-in amplifier

Figure 1a, b shows the experimental geometry of scanning photovoltage microscope. The laser is normally incident on the device, while a direct bias current is applied along the *x* axis (Fig. 1a). To investigate the effects of the laser helicity on the photovoltage generation, a photoelastic modulator (PEM) acting as a rotating quarter wave plate at a frequency of *f*_PEM_ ≈ 50 kHz is used to modulate the helicity of light. The magnitude of the helicity-dependent photovoltages (HDPs) is proportional to the difference between the photovoltages generated by left circularly polarized (LCP) and right circularly polarized light (RCP). The HDP is thus defined as *V*_±_ = *V*_RCP_ − *V*_LCP_. By employing PEM, a helicity-independent background signal due to the thermoelectric effect in TIs can be substantially suppressed in our HDP measurements.

In general, the HDPs appear in materials with spin-split band structures. When shining circularly polarized lights on the materials with strong spin orbit coupling normally, LCP and RCP light generate electrons with opposite spin direction. If no bias current is applied across the device, the absorption of LCP and RCP light is equal. Thus, the induced HDP is zero (*V*_±_ = *V*_RCP_ − *V*_LCP_). When applying bias currents, the current-induced out-of-plane spins accumulate near two opposing channel edges (Fig. 1a). As the amount of absorption of the LCP and RCP light changes depending on local spin accumulation due to current-induced effects, magnetic circular dichroism, which is a difference between the absorption of RCP and LCP light, is induced at the edges of the device channel. Therefore, the sign and magnitude of induced HDP provide information of the local spin polarization distribution.

### Scanning HDP data in Bi_2_Se_3_ and Pt

**Fig. 2:**
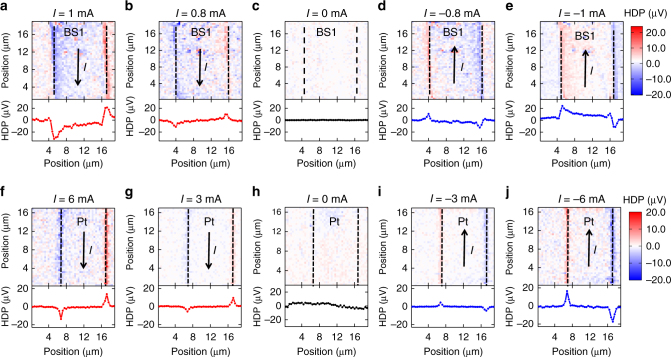
Current induced spin accumulations in Bi_2_Se_3_ and Pt. **a**–**e** Spatial two-dimensional HDP maps in BS1 under bias currents of 1 (**a**), 0.8 (**b**), 0 (**c**), −0.8 (**d**), and −1 mA (**e**). **f**–**j** Spatial two-dimensional HDP maps in Pt under bias currents of 6 (**f**), 3 (**g**), 0 (**h**), −3 (**i**), and −6 mA (**j**). Black dashed lines indicate the edges of the device. Black arrows show the applied bias current direction

Figure 2a–e shows the scanning HDP data in a Bi_2_Se_3_ channel (labeled as BS1) with different bias currents. The boundaries of the device channel are indicated by black dashed lines while black arrows show the direction of the applied bias current with the current density on the order of 10^6^ A cm^−2^. There is a negligible signal in the absence of bias currents in Fig. 2c. On the other hand, stronger signals are detected at the two edges of the device channel when the magnitude of bias currents increases. The signs of signals are opposite at two channel edges, and reversing the bias current direction causes the HDP to switch its sign. Considering the geometry of the electrical connections with the spin accumulation signals at edges of the device, we establish that the sign of the spin Hall angle *θ*_sh_ is positive in Bi_2_Se_3_, in good agreement with the previous results in TI/FM structures^35–38^.

In order to check whether our method can be applied to other material systems, HDP measurements are performed on a heavy metal Pt, which is known to have a strong current-induced spin accumulation with a positive sign of *θ*_sh_^17,42,44^. Figure 2f–j shows similar results as Bi_2_Se_3_, but with larger bias currents required to obtain similar HDP signal intensities due to overwhelming background conduction electrons in a metal. As a control experiment, we have performed measurements on Cu (Supplementary Fig. 4), which is known to have small spin orbit coupling. There is no observable signal in Cu either without or with bias current (Supplementary Fig. 4). We can also rule out the contribution from current-induced heating as reversing the current direction causes the HDP to switch its sign, indicating that our observed phenomena in Bi_2_Se_3_ and Pt are indeed current-induced spin accumulation due to sizable spin orbit interaction.

### Bias current and magnetic field dependence in Bi_2_Se_3_

**Fig. 3:**
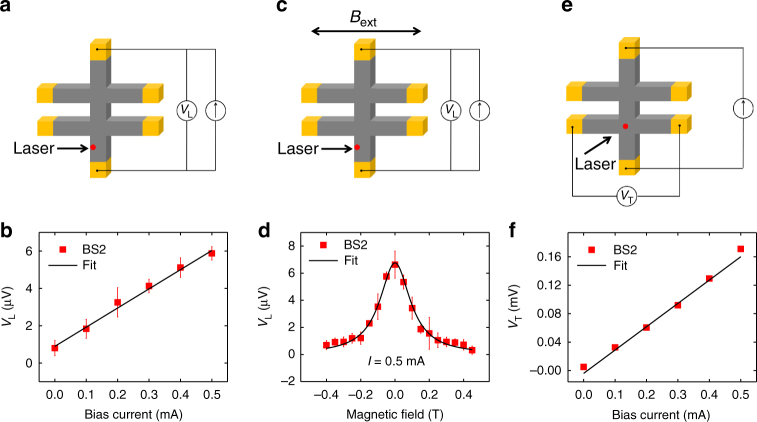
Current dependence of the spin accumulations in Bi_2_Se_3_. **a** Experimental geometry of longitudinal photovoltage (*V*_L_) measurement. **b** Bias current dependence of *V*_L_ by fixing the laser spot at one edge of the device in BS2. **c** Experimental geometry of Hanle measurement. **d** Measurement of HDP at one edge as a function of in-plane *B*_ext_ in BS2. **e** Experimental geometry of transverse photovoltage (*V*_T_) measurement. **f** Bias current dependence of *V*_T_ by fixing the laser spot at the center of the Hall cross in BS2. Solid lines are fits. The error bars in **b**, **d**, and **f** are the standard deviation from five measurements

We have further fixed the laser near one edge on another Bi_2_Se_3_ device (labeled as BS2) and measured the longitudinal HDP voltage (*V*_L_) while sweeping the bias current (Fig. 3a). As shown in Fig. 3b, the linear relationship of *V*_L_ with respect to the bias current suggests that the observed phenomenon in Bi_2_Se_3_ is indeed a current induced effect.

We have then performed Hanle measurements on BS2 to evaluate the spin lifetime. An in-plane external magnetic field (*B*_ext_) was applied perpendicular to the bias current direction to induce spin precession (Fig. 3c). Figure 3d shows the longitudinal HDP data obtained by locating the laser at one edge of the channel and sweeping the magnitude of the *B*_ext_. The data are fitted to $A_1/\left[ {1 + \left( {\Omega \tau _{\mathrm{s}}} \right)^2} \right]$, where *A*_1_ is the peak of HDP, $\Omega = g\mu _{\mathrm{B}}B_{{\mathrm{ext}}}/\hbar$, *g* is *g*-factor, *μ*_B_ is the Bohr magneton, and $\hbar$ is reduced Plank constant^6^. The spin lifetime (*τ*_s_) of Bi_2_Se_3_ is evaluated to be ~3.3 ± 0.13 ps (Supplementary Note 2), which is similar to the reported values in the previous studies^30,45–47^.

### Extraction of spin Hall angle in Bi_2_Se_3_ and the role of bulk spin Hall effect

We next evaluate the spin Hall angle *θ*_sh_ in BS2, where the HDP is detected along the transverse direction (*V*_T_, Fig. 3e) with respect to the bias current^48^. In our spin Hall angle evaluation method, circularly polarized light incident on the Hall bar device perpendicular to the sample plane excites out-of-plane spin-polarized carriers, which generate a spin-dependent transverse voltage *V*_T_ due to inverse spin Hall effect^48,49^. *V*_T_ can be written as $V_{\mathrm{T}} = \theta _{{\mathrm{sh}}}\rho _{\mathrm{N}}wJ_{//}P$, where *θ*_sh_ is spin Hall angle, *ρ*_N_ is the resistivity of the sample, *w* is the channel width, *J*_// _is the bias current density, and *P* is the carrier spin polarization. We obtain the spin Hall angle *θ*_sh_ ~ 0.0085 ± 0.0016 (Supplementary Note 3), in line with previous findings of *θ*_sh_ in Bi_2_Se_3_^37,38^. Recent experiments using various transport methods demonstrated a wide range of the charge-to-spin conversion efficiency in Bi_2_Se_3_ from 0.009 to 3.5^35–38,50,51^. The *θ*_sh_ measured in our work is similar to the value obtained from spin pumping, indicating that the spin currents generated from the bulk contributes significantly to our observed signal^37,38^.

We further discuss the contribution of topological surface states (TSS) and BS to the out-of-plane edge spin accumulation in Bi_2_Se_3_. We can rule out the two-dimensional electron gas (2DEG) states as the major contribution for a spin accumulation since the 2DEG provides the opposite spin polarity compared to the observed spin accumulation direction in Fig. 2a–e^52–54^. By employing the multi-channel model as reported previously^55,56^, the ratio of the spin current flowing in TSS and BS is *I*_s*-*TSS_:*I*_s-BS_ = 1:2.06 considering the thickness of TSS to be ~1 nm and BS to be ~1.9 nm (Supplementary Note 4)^52,56,57^, indicating a considerable contribution of the bulk spin Hall effect in 9 quintuple layer (QL) Bi_2_Se_3_. We perform the thickness-dependent study on Bi_2_Se_3_. The HDP magnitude increases significantly when *t*_BiSe_ < 9 QL as shown in Supplementary Fig. 5, indicating a considerable bulk spin Hall effect in 9 QL Bi_2_Se_3_ at room temperature (Supplementary Note 4).

### HDPs in BiSbTeSe_2_

**Fig. 4:**
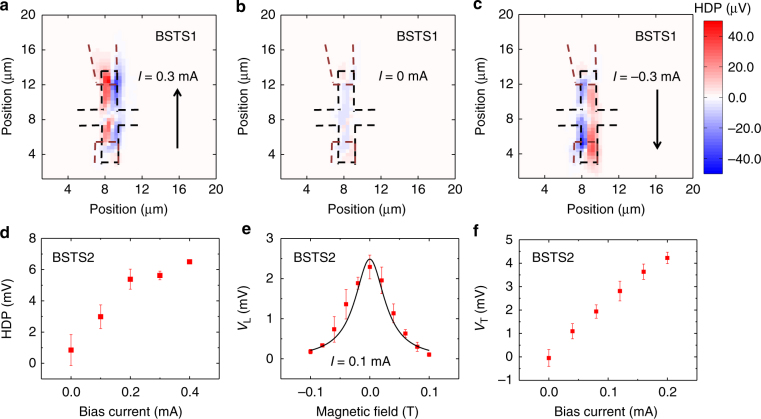
Current induced spin accumulations in BiSbTeSe_2_. **a**–**c** Spatial two-dimensional HDP map in BSTS1 under bias currents of 0.3 (**a**), 0 (**b**), −0.3 mA (**c**) with *I*_pump_ = 0.4 mW. Black dashed lines represent the boundaries of the device. Black arrows show the direction of applied bias current. **d** Bias current dependence of *V*_L_ by fixing the laser spot at one edge of the device in BSTS2. **e** Measurement of HDP at one edge as a function of *B*_ext_ in BSTS2. **f** Bias current dependence of *V*_T_ by fixing the laser spot at the center of the Hall cross in BSTS2. Solid lines are fits. The error bars in **d**, **e**, and **f** are the standard deviation from five measurements

We have performed similar measurements on BiSbTeSe_2_ (labeled as BSTS1), which was reported to have a larger spin-charge conversion efficiency. As shown in Fig. 4a, c, the spin polarization has opposite signs for the two edges and changes the sign on reversing the current direction, similar to the data from Bi_2_Se_3_. In contrast, there is a negligible signal in the absence of bias currents in Fig. 4b. Under the presence of bias currents, the *V*_L_ is proportional to the bias current (Fig. 4d), indicating the current-induced effect. Furthermore, we have performed the magnetic-field dependent study (Fig. 4e) on BiSbTeSe_2_ by fixing the laser near one edge of another device (BSTS2). The data are fitted to the Hanle equation and the spin lifetime is extracted to be ~18.6 ± 1.5 ps (Supplementary Note 2). We then measure the *V*_T_ in BSTS2 (Fig. 4f), and the spin Hall angle in BSTS2 is evaluated to be ~0.0616 ± 0.0101 (Supplementary Note 3). We further calculate the spin current flowing in TSS and BS by employing the multi-channel model. The ratio of the spin current flowing in TSS to BS spin current is *I*_s-TSS_:*I*_s-BS = _1:1.62, indicating a sizable bulk contribution at room temperature. In line with the above results, the Fermi level of BSTS2 is estimated to be located inside the bulk conduction band (Supplementary Note 5)^58^.

## Discussion

We have shown that the current induced spin accumulation not only in TIs but also in heavy metals (Supplementary Notes 6 and 7 and Supplementary Figs. 6 and 7) can be detected and imaged by a helicity-dependent scanning photovoltage setup at room temperature. The required sample structure is free from a FM, thus eliminating the interface transparency issue and current shunting problem in a bilayer structure, which typically exist in other electrical characterization methods. The accumulated spin directions at channel edges, the spin lifetime, and the spin Hall angle can be estimated using this technique, which propels the characterization of novel materials and helps for a better understanding of the interaction among charge, spin, and light.

We would like to note that while we were preparing the revised manuscript, we became aware of a similar work by other group^59^.

## Methods

### Sample and device fabrication

We employ a previously reported two-step procedure to grow 10 QL (1 QL ≈ 1 nm) Bi_2_Se_3_ on Al_2_O_3_ (0001) substrates^56,60^. The high-quality molecular beam epitaxy (MBE) grown Bi_2_Se_3_ thin film was then patterned into Hall bar devices (Fig. 1b) by the following processes. First, the Se layer was decapped by annealing in the vacuum chamber. Then, we etched the film by Ar-ion milling for 2 s and a MgO (1 nm)/SiO_2_ (3 nm) capping layer was sputtered onto the Bi_2_Se_3_ film at room temperature with a base pressure of 3 × 10^−9^ Torr. The final thickness of the Bi_2_Se_3_ film is 9 nm. Then the film was patterned into a Hall bar by photolithography and Ar ion milling. The dimension of the Hall bar device is 450 µm in the longitudinal direction, 100 µm in the transverse direction, and the bar width is 10 µm. In the next step, a top electrode of Ta (5 nm)/Cu (100 nm)/Ru (5 nm) was deposited. The Bi_2_Se_3_ device characterization is shown in Supplementary Note 1 and Supplementary Fig. 2.

High-quality BiSbTeSe_2_ (BSTS) single crystals were grown by the modified Bridgeman technique. We exfoliated ~100–150-nm-thick BSTS nanoflakes onto the n-doped Si wafers with a 300-nm-thick thermal SiO_2_ layer. A MgO (1 nm)/SiO_2_ (3 nm) capping layer was sputtered. The Hall bar devices (Supplementary Fig. 3a) were patterned by electron beam lithography followed by Ar-ion milling. The device dimension is 30 µm in the longitudinal, 10 µm in the transverse direction, and the bar width is 4 µm. Then a top electrode of Ta (5 nm)/Cu (100 nm)/Ru (5 nm) was deposited. The BiSbTeSe_2_ device characterization is shown in Supplementary Note 1 and Supplementary Fig. 3.

The 6-nm-thick Pt thin film was deposited by magnetron sputtering. A MgO (1 nm)/SiO_2_ (3 nm) capping layer was then sputtered on top of Pt thin film. The Hall bar device was patterned by standard photolithography and Ar-ion milling. The device dimension is 450 µm in the longitudinal, 100 µm in the transverse direction and the bar width is 10 µm. Finally, the top electrode of Ta (5 nm)/Cu (100 nm)/Ru (5 nm) was deposited. We repeated the same fabrication process to fabricate a 6-nm-thick copper Hall bar device for the control experiment.

### Measurements

In all experiments (unless otherwise indicated), laser light (wavelength *λ* = 650 nm, power of pump laser beam *I*_pump_ = 4.62 mW) was focused at normal incidence onto bare Bi_2_Se_3_, BiSbTeSe_2_ or Pt using a 100× microscope objective lens (Supplementary Fig. 1). The laser spot size is ~1 µm and the spatial resolution is set by the size of laser spot. The laser beam was split into two after reflecting from the sample; one was collected by a CMOS detector to display the position of the laser spot on the device, while the other one was detected by a photodetector to measure the reflectivity of the device. Two-dimensional image was achieved by scanning the device with the laser spot while moving the device with a piezo stage.

To measure the HDP, the light circular polarization was modulated while the light intensity was constant. Light circular polarization was modulated using a PEM. In this configuration, the PEM acts as a rotating quarter wave plate and modulates the polarization of the laser at a fixed frequency of 50 kHz and without affecting the laser intensity. The angle of light linear polarization before the PEM was set to 45° with respect to the axis of the PEM. The HDP between the device electrodes was acquired by a lock-in amplifier.

### Data availability

The data that support the plots within this paper and other findings of this study are available from the corresponding author upon reasonable request.

## Electronic supplementary material

Supplementary Information
