# Supplementary material for: Direct visualization of current-induced spin accumulation in topological insulators
Source: Nat Commun. 2018 Jun 27;9:2492. doi: 10.1038/s41467-018-04939-6 (PMC6021425; doi:10.1038/s41467-018-04939-6)
Supplement: Supplementary file 1 — Supplementary Information [file 41467_2018_4939_MOESM1_ESM.pdf]

# Direct visualization of current induced spin accumulation in topological insulators

**Liu et al.**

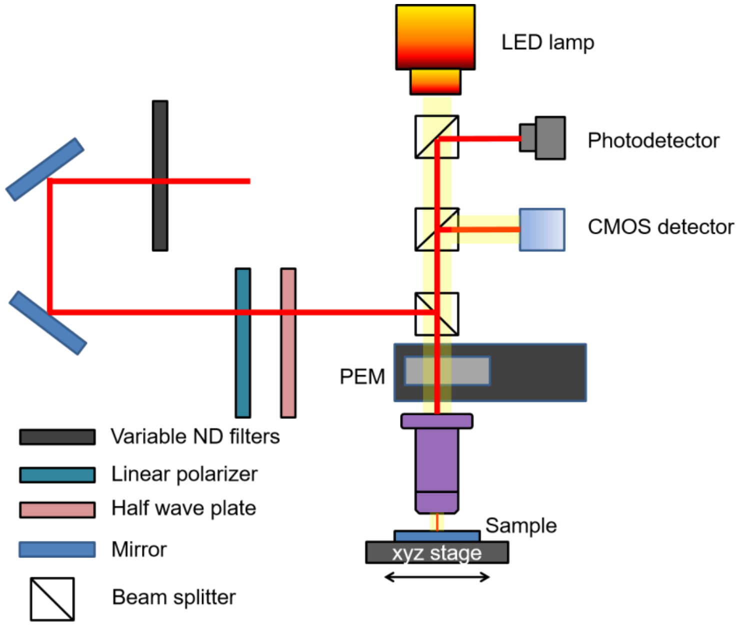


**Supplementary Figure 1 | Schematics of experimental setup.** The focused laser is normally incident on the sample. The reflected laser beam was split into two; one is collected by a CMOS detector to display the position of laser spot on the device, while the other one is detected by a photodetector to measure the reflectivity of the device. Two dimensional image is achieved by scanning the device with the laser spot while moving the device with a piezo stage.

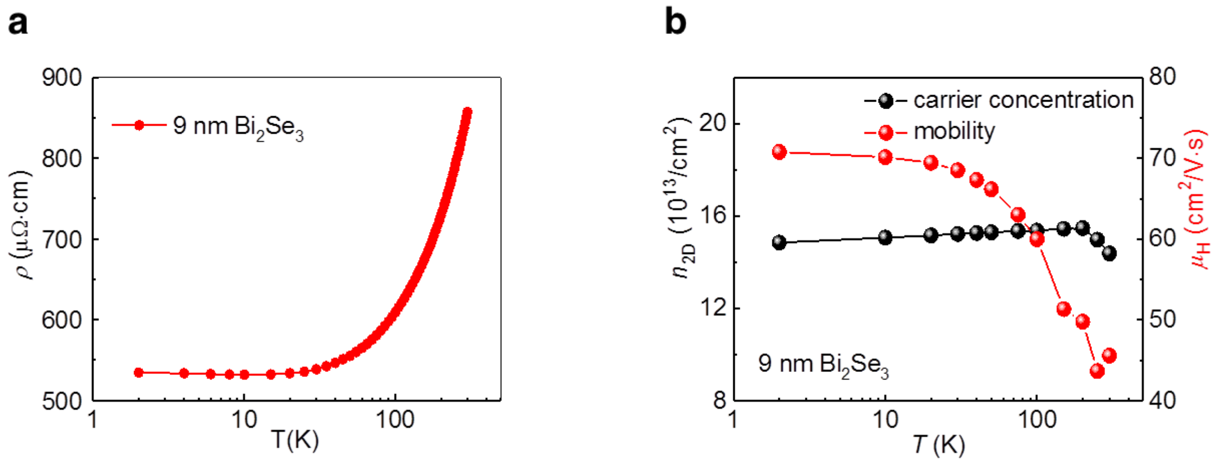


**Supplementary Figure 2 | $Bi_2Se_3$ device characterization.** **a**, Temperature dependence of the resistivity. **b**, Carrier concentration and mobility of 9 QL $Bi_2Se_3$.

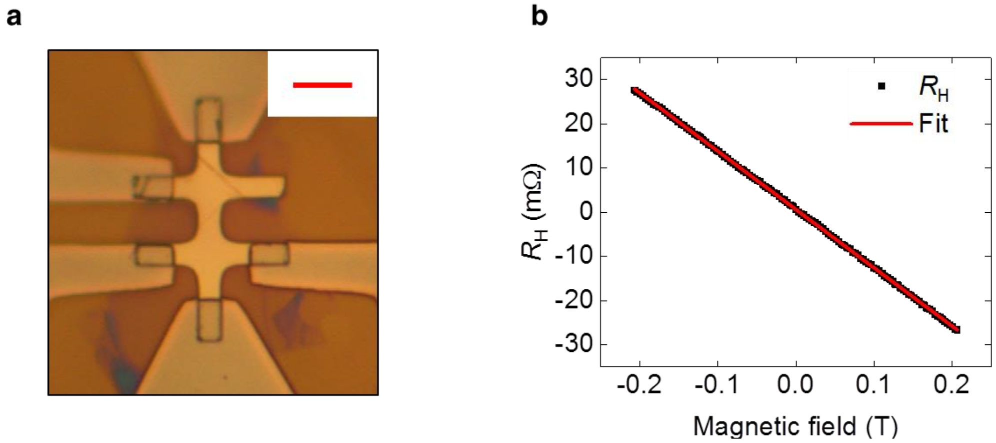


**Supplementary Figure 3 | $BiSbTeSe_2$ device characterization. a,** Optical image of BSTS device. Scale bar is 10 μm. **b,** Hall resistance curve with a linear fit for BSTS at room temperature.

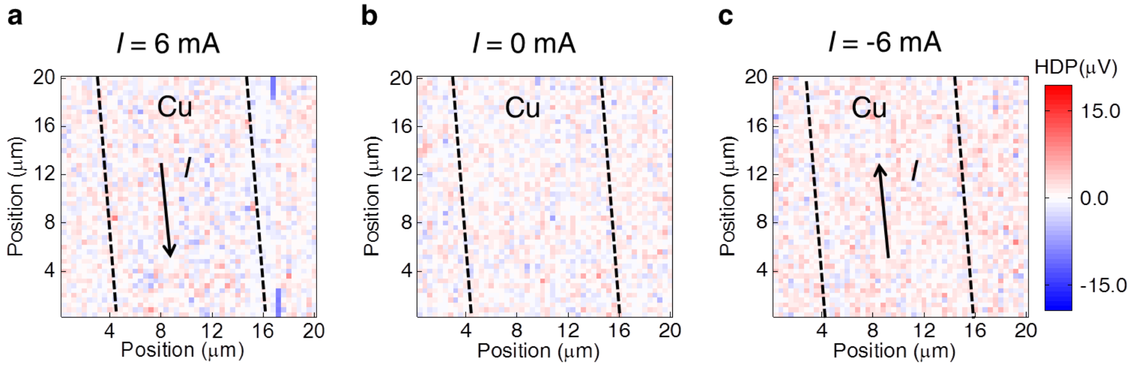


**Supplementary Figure 4 | Current induced spin accumulations in Cu. a-c,** Spatial two-dimensional HDP maps in Cu under bias currents of 6 (**a**), 0 (**b**), −6 mA (**c**). There is no observable signal either without or with bias currents.

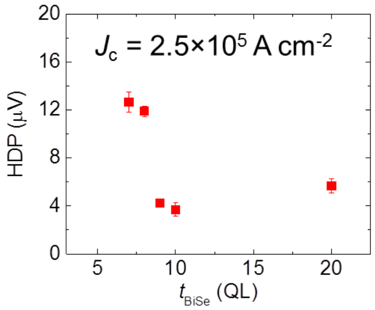


**Supplementary Figure 5 | The HDP as a function of $Bi_2Se_3$ ($t_{BiSe}$) thickness at room temperature.** Each HDP represents the average value from five measurements. The error bars are the standard deviation from five measurements.

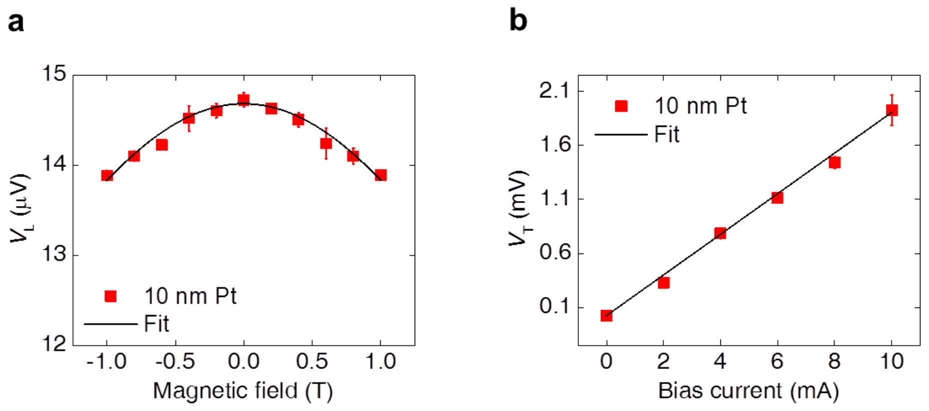


**Supplementary Figure 6 | Hanle measurement (a) and transverse voltage ($V_T$) measurement (b) in 10 nm Pt.** The error bars are the standard deviation from five measurements.

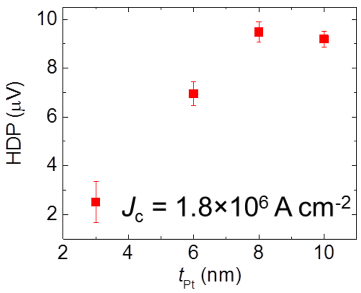


**Supplementary Figure 7 | The HDP as a function of Pt thickness ($t_{Pt}$) at room temperature.** The error bars are the standard deviation from five measurements.

**Supplementary Note 1. Materials characterization**

The temperature dependent resistivity, carrier concentration and mobility of 9-nm-thick $Bi_2Se_3$ film were obtained from four probe and Hall measurements. As shown in Supplementary Fig. 2a, the almost constant sheet resistivity when the temperature is below 30K suggests the surface dominated transport behavior, which is a typical characteristic of $Bi_2Se_3$. Supplementary Fig. 2b indicates that our sample is electron doped with a sheet carrier concentration ($n_{2D}$) of $n_{2D}$ ~ 14.3 × $10^{13}$ $cm^{-2}$ and carrier mobility ($\mu$) of $\mu$ ~ 45.5 $cm^2$ $V^{-1}$ $s^{-1}$ at room temperature.

We then performed Hall measurements on $BiSbTeSe_2$ sample. As shown in Supplementary Fig. 3b, the negative Hall slope indicates that our BSTS sample is electron doped. The sheet carrier concentration at room temperature is determined to be ~ 4.8 × $10^{13}$ $cm^{-2}$, which is in a similar range compared with previous studies[1,2].

**Supplementary Note 2. Hanle analysis**

The Hanle effect denotes the precession of spins when applying an external magnetic field ($B_{ext}$) perpendicular to the spin direction. The frequency of the spin precession is known as Larmor frequency $\Omega = \mu_B g B/\hbar$, where $\mu_B$ is Bohr magneton, *g* is the *g*-factor, *B* is the magnetic field and $\hbar$ is reduced Plank constant. The time evolution of the electron spin is given by $S_0 \cos\Omega t \exp\left(-t/\tau_s^r\right)$ with $S_0$, $\Omega$, $t$ and $\tau_s^r$ being the initial amount of spins, the Larmor precession rate, time and spin relaxation time, respectively. Averaging this spin direction across time and considering the electron life time gives $S = \int_0^\infty dt W(t) S_0 \cos\Omega t \exp\left(-t/\tau_s^r\right)$, where $W(t) = \tau^{-1}\exp(-t/\tau)$ under thermodynamic equilibrium and $\tau$ is the electron lifetime. By

introducing $1/\tau_{\mathrm{s}} = 1/\tau + 1/\tau_{\mathrm{s}}^{\mathrm{r}}$, along with the abovementioned equations, we can get the well-known expression for the Hanle analysis, $S(B_{\mathrm{ext}}) = S_0 / \left( (\Omega \tau_{\mathrm{s}})^2 + 1 \right)$, which give the basis for determining the spin lifetime of materials. In the case of $Bi_2Se_3$, the *g*-factor is measured to be ~ 23 from previous studies[3]. Thus, the spin lifetime $\tau_{\mathrm{s}}$ can be estimated to be ~ 3.3 ± 0.13 ps. While in the case of $BiSbTeSe_2$, the spin lifetime $\tau_{\mathrm{s}}$ is estimated to be 18.6 ± 1.5 ps by using $g$ = 20 [Ref. 4].

**Supplementary Note 3. Spin Hall angle estimation in $Bi_2Se_3$ and $BiSbTeSe_2$**

The spin Hall angle is defined as $\theta_{\mathrm{sh}} = \sigma_{\mathrm{sh}} / \sigma_{\mathrm{N}}$, where $\sigma_{\mathrm{sh}}$ and $\sigma_{\mathrm{N}}$ are the spin Hall conductivity and device charge conductivity, respectively. The transverse electric field generated by ISHE can be written as $\mathbf{E}_{\perp} = \rho_{\mathrm{sh}} (\mathbf{J_s} \times \boldsymbol{\sigma}) = \theta_{\mathrm{sh}} \rho_{\mathrm{N}} (\mathbf{J_s} \times \boldsymbol{\sigma})$, where $\mathbf{J_s}$ is the spin current density and $\boldsymbol{\sigma}$ is the spin-polarization unit vector. The spin current density can be further replaced by $\mathbf{J_s} = \mathbf{J}_{//} P$, where $\mathbf{J}_{//}$ is the bias current density and $P$ is the carrier spin polarization. Finally, the magnitude of transverse electric field can be substituted by $E_{\perp} = V_{\mathrm{T}} / w$ and thus $V_{\mathrm{T}} = \theta_{\mathrm{sh}} \rho_{\mathrm{N}} w J_{//} P$, where $w$ is the channel width.

In order to estimate the spin Hall angle in $Bi_2Se_3$, we need to get the carrier spin polarization ($P$), which can be expressed as $P = G P_0 \tau_{\mathrm{s}} / n_{\mathrm{e}}$ with the spin lifetime $\tau_{\mathrm{s}}$, initial spin-polarization $P_0$, the optically excited electron density rate $G$, and the total electron density $n_{\mathrm{e}}$. The spin lifetime $\tau_{\mathrm{s}}$ can be estimated to be ~ 3.3 ± 0.13 ps from Hanle measurements (Fig. 3d). The optically excited electron density can be written as $G = \alpha n_{\mathrm{p}} (1 - R) / d \int_0^d e^{-\alpha z} dz$ with $\alpha$ is the

absorption coefficient, $n_p$ is the irradiated photon density per unit time, $R$ is the reflectance of the sample, $d$ is the sample thickness and $z$ is the variable of the sample thickness direction. We take $R$ = 0.30 and $\alpha = 1.05 \times 10^6$ cm$^{-1}$ in a 10 nm $Bi_2Se_3$ thin film reported from the previous studies[4]. The irradiated photon density per unit time is calculated to be $4.81 \times 10^{23}$ cm$^{-2}$. The photoexcited spin polarized carrier density can be estimated as $n \sim I\alpha\tau/h\nu = 1.01\times10^{25}$ m$^{-3}$, where $I$ is the laser intensity. The initial out-of-pane spin polarization $P_0$ in $Bi_2Se_3$ is estimated to be ~ 0.1.[5] Thus the carrier spin polarization $P$ can be calculated to be $(7.10 \pm 0.28) \times 10^{-3}$. The transverse voltage $V_T$ is 0.168 ± 0.03 mV under a bias current of 0.5 mA (Fig. 3f). Thus, the spin Hall angle $\theta_{sh}$ is calculated to be ~ 0.0085 ± 0.0016.

In the case of $BiSbTeSe_2$, the spin lifetime is calculated to be 18.6 ± 1.5 ps from the Hanle measurements (Fig. 4e) and the resistivity is decided to be $1.6 \times 10^{-4}$ Ω·m from transport measurements. The absorption coefficient and reflectance in $BiSbTeSe_2$ are $\alpha = 2 \times 10^4$ cm$^{-1}$ and $R$ = 0.21, respectively. We can get $n \sim 1.79 \times 10^{23}$ m$^{-3}$. We use the initial spin polarization value $P_0$ ~ 0.25 to estimate the carrier spin polarization $P$[6]. The transverse voltage $V_T$ is 2.8 ± 0.4 mV under a bias current of 0.12 mA (Fig. 4f). The spin Hall angle in $BiSbTeSe_2$ can be estimated to be ~ 0.0616 ± 0.0101.

**Supplementary Note 4. The contribution of TSS and BS to spin accumulation in $Bi_2Se_3$**

We first discuss the contribution of TSS, 2DEG and BS to the out-of-plane accumulation in 9 QL $Bi_2Se_3$. We employ the multi-channel model to estimate the spin current generated from topological surface states (TSS), 2DEG and bulk states (BS). The thickness of TSS and 2DEG in $Bi_2Se_3$ was reported to be ~ 1 nm and ~ 4 nm, respectively[7,8,9]. The estimated spin diffusion length in 9 QL $Bi_2Se_3$ is estimated ~ 1.9 ± 0.39 nm by using the relationship $\lambda = \sqrt{D\tau_s}$ where $D$

is the spin diffusion coefficient and $\tau_s \sim 3.3 \pm 0.13$ ps is the spin lifetime. We take $D \sim 1.2 \times 10^{-2}$ $cm^2/s$ extracted from the electron mobility using $D = \mu k_B T / e$, where $\mu$ is the electron mobility, $k_B$ is the Boltzmann constant, $T$ is the temperature.

In order to estimate the charge current flowing in each state, we need to know the conductance in TSS, 2DEG and BS. Recent measurements of $Bi_2Se_3$ conductance by terahertz time-domain spectroscopy suggest that the conductance of TSS, 2DEG and BS in 9 QL $Bi_2Se_3$ are ~ $2G_0$, $6G_0$ and $10G_0$, respectively, where $G_0 = e^2/h$ denotes single conductance quantum[9]. As a result, the ratio of the charge current flowing in TSS, 2DEG and bulk is $I_{TSS}:I_{2DEG}:I_{bulk}$ = 1:3:5. The ratio of current density flowing in the three states is thus $J_{TSS}:J_{2DEG}:J_{bulk}$ = 7.6:5.7:20 (the channel width is the same for the TSS, 2DEG and bulk). Next we calculate the spin current $J_s = \theta_{sh} J_c$ flowing in TSS, 2DEG and BS by considering the spin Hall angle in respective state.

The contribution from 2DEG shows the opposite spin polarity with respect to our observed spin accumulation distribution[7,10,11]. We estimate the relative contribution of TSS and Rashba states by comparing the amplitude of spin splitting $\hbar v_F/\alpha \sim 9.1$, suggesting that the Rashba state is the not the main contribution of the current induced spin accumulation in the 9 QL sample. Furthermore, to our best knowledge, the Rashba induced spin accumulation in TIs is along the in-plane direction. Thus, whether the Rashba states can generate out-of-plane spins is not clear at this moment in the community, and future works are necessary. We thus only consider the contribution of TSS and BS.

According to recent reported spin Hall angles measured by ST-FMR and spin pumping, the spin Hall angle ($\theta_{sh}$) originated from TSS is between 0.047 and 3.5[12,13] while $\theta_{sh}$ originated from bulk states is between 0.0093 and 0.43[14,15]. For simplicity, we consider $\theta_{sh}$ originated from TSS and bulk states with 0.047[13] and 0.01944[15], respectively. Thus, the ratio of spin current

density flowing in TSS to that in BS is estimated to be $J_{s\text{-}TSS}$:$J_{s\text{-}BS}$ = 3.57:3.88. We then convert the spin current density to the spin current by considering the thickness of the TSS and BS. The ratio of the spin current flowing in TSS to that in BS is estimated to be $I_{s\text{-}TSS}$:$I_{s\text{-}BS}$ = 1:2.06, indicating that there is a considerable bulk spin Hall contribution to the spin accumulation in 9 QL $Bi_2Se_3$ at room temperature.

We have then performed the thickness dependence study on $Bi_2Se_3$ at room temperature. As shown in Supplementary Fig. 5, HDP shows a relatively constant value ~ 4 μV for 9, 10 and 20 QL devices, and starts to increase below 9 QL, reaching a maximum of ~ 12.6 μV at 7 QL. Previous reports showed that negligible bulk states are expected when the thickness of $Bi_2Se_3$ is below 8 QL[16]. We thus attribute the HDP measured on 7 QL and 8 QL to TSS. While in the case of 9, 10 and 20 QL devices, the smaller HDP signal could be due to a considerable bulk state contribution.

**Supplementary Note 5. The contribution of TSS and BS to spin accumulation in $BiSbTeSe_2$**

The thickness of TSS in $BiSbTeSe_2$ was reported to be ~ 2.5 nm[17]. The estimated spin diffusion length in BSTS2 is estimated ~ 5.82 ± 1.67 nm. In order to estimate the charge current flowing in TSS and BS, we use the carrier concentration to estimate the current flowing in TSS and BS by employing $I_{TSS} = n_{TSS}\mu_{TSS}eEW$ and $I_{BS} = n_{BS}\mu_{BS}eEW$ where $W$ and E are the channel width and electric field (same for TSS and BS), respectively. We assume $\mu_{TSS} = \mu_{BS}$ since the linearity of the Hall curve as shown in Supplementary Fig. 3. The carrier concentration of bulk and surface was calculated using the model reported previously[18]. The ratio of the bulk to surface carrier concentration in $BiSbTeSe_2$ at room temperature is estimated to be ~ 3:1. As a result, the ratio of the charge current flowing in TSS and BS is $I_{TSS}$:$I_{bulk}$ = 1:3. The ratio of current density

flowing in TSS and BS is thus $J_{TSS}$:$J_{bulk}$ = 5.82:7.5 (the channel width is the same for the TSS and bulk). Next we calculate the spin current $J_s = \theta_{sh} J_c$ flowing in TSS and BS by considering the spin Hall angle in respective state. For simplicity, we consider $\theta_{sh}$ originated from TSS and bulk states with 0.01[19] and 0.0054[18], respectively. Thus, the ratio of spin current flowing in TSS over BS is estimated to be ~ 1:1.62, indicating a substantial contribution of bulk $BiSbTeSe_2$ at room temperature. We further estimate the Fermi level $E_F$ in BSTS2 by the carrier concentration from Hall measurements[18]. Taking the Dirac point as $E = 0$, the $E_F$ in $BiSbTeSe_2$ is estimated to be ~ 0.25 eV. Since the bottom of the conduction band is at $E_{CB} \approx 0.21$ eV, the Fermi level of our device is located inside the conduction band, $E_F - E_{CB} \approx 0.04$ eV.

**Supplementary Note 6. Spin Hall angle estimation in Pt**

In order to estimate the spin Hall angle in Pt, we first performed Hanle measurements and the result is shown in Supplementary Fig. 6a. The spin lifetime in Pt can be estimated to be ~ 0.57 ± 0.035 ps by setting the *g*-factor to 4.9 [Ref. 20]. The resistivity in our sample is measured to be 33 μΩ·cm from transport measurements. The absorption coefficient of $\alpha = 8.21 \times 10^5$ cm$^{-1}$ [Ref. 21] and reflectivity $R = 0.32$. The photoexcited spin polarized carrier density can be estimated as $n \sim 2.25 \times 10^{23}$ m$^{-3}$. The spin polarization $P$ can be estimated by considering $P_0 \sim 0.30$ from a previous study[21]. The transverse voltage $V_T$ is 0.78 ± 0.023 mV under a bias current of 4 mA (Supplementary Fig. 6b). The spin Hall angle in Pt is thus estimated to be ~ 0.0085 ± 0.0006.

## Supplementary Note 7. Thickness dependence of Pt

By carrying out the thickness dependence study, we show that the generated photovoltage increases when increasing the thickness of Pt to 8 nm and saturates when the thickness of Pt is above 8 nm as shown in Supplementary Fig. 7. We thus conclude that the bulk spin Hall effect is dominant to the photovoltage on Pt in our study.

## Supplementary References


1. Wang, W. *et al.* Intrinsic topological insulator $Bi_{1.5}Sb_{0.5}Te_{3-x}Se_x$ thin crystals. *Sci. Rep.* **5**, 7931 (2015).
2. Banerjee, K. *et al.* Defect-induced negative magnetoresistance and surface state robustness in the topological insulator $BiSbTeSe_2$. *Phys. Rev. B* **90**, 235427 (2014).
3. Köhler, H. & Wöchner, E. The g-factor of the conduction electrons in $Bi_2Se_3$. *Phys. Status Solidi B* **67**, 665-675 (1975).
4. Glinka, Y. D. *et al.* Ultrafast carrier dynamics in thin-films of the topological insulator $Bi_2Se_3$. *Appl. Phys. Lett.* **103**, 151903 (2013).
5. Jozwiak, C. *et al.* Widespread spin polarization effects in photoemission from topological insulators. *Phys. Rev. B* **84**, 165113 (2011).
6. Souma, S. *et al.* Direct measurement of the out-of-plane spin texture in the Dirac-cone surface state of a topological insulator. *Phys. Rev. Lett.* **106**, 216803 (2011).
7. Bahramy, M. S. *et al.* Emergent quantum confinement at topological insulator surfaces. *Nat. Commun.* **3**, 1159 (2012).
8. Bansal, N., Kim, Y. S., Brahlek, M., Edrey, E. & Oh, S. Thickness-independent transport channels in topological insulator $Bi_2Se_3$ thin films. *Phys. Rev. Lett.* **109**, 116804 (2012).
9. Park, B. C. *et al.* Terahertz single conductance quantum and topological phase transitions in topological insulator $Bi_2Se_3$ ultrathin films. *Nat. Commun.* **6**, 6552 (2015).
10. Wray, L. A. *et al.* A topological insulator surface under strong Coulomb, magnetic and disorder perturbations. *Nat. Phys.* **7**, 32 (2010).
11. King, P. D. C. *et al.* Large tunable Rashba spin splitting of a two-dimensional electron gas in $Bi_2Se_3$. *Phys. Rev. Lett.* **107**, 096802 (2011).
12. Mellnik, A. R. *et al.* Spin-transfer torque generated by a topological insulator. *Nature* **511**, 449-451 (2014).
13. Wang, Y. *et al.* Topological surface states originated spin-orbit torques in $Bi_2Se_3$. *Phys. Rev. Lett.* **114**, 257202 (2015).
14. Deorani, P. *et al.* Observation of inverse spin Hall effect in bismuth selenide. *Phys. Rev. B* **90**, 094403 (2014).
15. Jamali, M. *et al.* Giant spin pumping and inverse spin Hall effect in the presence of surface and bulk spin−orbit coupling of topological insulator $Bi_2Se_3$. *Nano Lett.* **15**, 7126-7132 (2015).
16. Wang, Y. *et al.* Room temperature magnetization switching in topological insulator-ferromagnet heterostructures by spin-orbit torques. *Nat. Commun.* **8**, 1364 (2017).

17. Xia, B. *et al.* Indications of surface-dominated transport in single crystalline nanoflake devices of topological insulator $Bi_{1.5}Sb_{0.5}Te_{1.8}Se_{1.2}$. *Phys. Rev. B* **87**, 085442 (2013).
18. Yang, F. *et al.* Switching of charge-current-induced spin polarization in the topological insulator $BiSbTeSe_2$. *Phys. Rev. B* **94**, 075304 (2016).
19. Shiomi, Y. *et al.* Spin-electricity conversion induced by spin injection into topological insulators. *Phys. Rev. Lett.* **113**, 196601 (2014).
20. Gustafsson, P., Ohlsén, H. & Nordborg, L. Conduction-electron g-factor measurements in platinum. *Phys. Rev. B* **33**, 3749-3755 (1986).
21. Garbe, J., Venus, D., Suga, S., Schneider, C. & Kirschner, J. Spin-polarized angle-resolved photoemission from the (110) surface of platinum. *Surf. Sci.* **178**, 342-348 (1986).
